# Typical Hus: Evidence of Acute Phase Complement Activation from a Daycare Outbreak

**DOI:** 10.21767/2472-5056.100011

**Published:** 2016-05-06

**Authors:** Tammy M Brady, Cozumel Pruette, Lauren F Loeffler, Darcy Weidemann, John J Strouse, Eleni Gavriilaki, Robert A Brodsky

**Affiliations:** 1 Division of Pediatric Nephrology, Johns Hopkins University School of Medicine, USA; 2 Division of Pediatric Nephrology, Children’s Mercy Hospital, Kansas City, MO, USA; 3 Division of Pediatric Hematology Johns Hopkins University School of Medicine, USA; 4 Division of Hematology, Johns Hopkins University School of Medicine, USA

**Keywords:** Hemolytic uremic syndrome, Hemolytic anemia, White blood cell

## Abstract

The clinical manifestations of typical hemolytic uremic syndrome (HUS) encompass a wide spectrum. Despite the potentially severe sequelae from this syndrome, treatment approaches remain supportive. We present the clinical course of a child who contracted Shiga toxin-positive *E. coli* (STEC) from a daycare center during an outbreak. Utilizing the modified Ham test which is a rapid, serum-based functional assay used to detect activation of the alternative pathway of complement as observed in atypical HUS, patient sera revealed evidence of increased complement activation in the acute phase of the syndrome but not after resolution. Further, this complement activation was attenuated by eculizumab *in vitro*, an effect that was replicated *in vitro* utilizing Shiga toxin as a stimulus of complement activation in normal serum. Our report suggests that complement blockade may be effective in the treatment of STEC-HUS when initiated early in the disease. Given the epidemic nature of the disease that limits the feasibility of randomized clinical trials, further studies are needed to determine the value of early eculizumab treatment in STEC-HUS.

## Introduction

The clinical manifestations of Shiga toxin-positive *E. coli* hemolytic uremic syndrome (STEC-HUS) span a wide spectrum, with some children mildly affected with minimal symptoms to other children having a severe clinical course resulting in renal failure, substantial neurological consequences, and even death. The care of children with STEC-HUS remains supportive, with no therapies available to directly treat the underlying pathophysiology. Evidence from human [1,2] and animal [3,4] models have suggested that complement activation may play a role in the course of STEC-HUS, although this has not been fully characterized. Eculizumab, a C5 monoclonal antibody indicated for the treatment of atypical HUS (microangiopathic hemolytic anemia due to complement dysregulation as a result of acquired or genetic disorders[5]), has been used in STECHUS with reports suggesting some benefit [6,7] and others stating no benefit [8,9]. In this report, we present the case of a child who contracted STEC from a daycare center during an outbreak. Sera provided for testing revealed evidence of increased complement activation in the acute phase of the syndrome.

## Case Report

The child is a 28 month old previously healthy white male who developed bloody diarrhea after three days of fever, crampy abdominal pain, emesis, and non-bloody diarrhea. Initial evaluation was notable for white blood cell count (WBC) 14.8 k/mm^3^, hemoglobin 13 g/dL, platelets 279 k/mm3, creatinine 0.3 mg/dL and a physical exam concerning for intussusception. Abdominal ultrasound was notable for colitis. During hospital admission, he received intravenous fluid and morphine for pain control. He became oliguric, with acute renal failure, anemia and thrombocytopenia noted on hospital day (HD) 3: creatinine 3.1 mg/dL, platelets 9 k/mm3, hemoglobin 5.8 g/dL. A peripheral blood smear revealed schistocytes and severely decreased platelets, consistent with a microangiopathic hemolytic anemia. His renal failure progressed and peritoneal dialysis (PD) was initiated on HD 5 and continued for seven days. His peak creatinine was 5.7 mg/dL, which decreased to 0.6 mg/dL by discharge (HD 15). He required six packed red blood cell (PRBC) transfusions to maintain hemoglobin >6 g/dL and received two platelet transfusions, each prior to a procedure. Three days after discharge his hemoglobin was 12 g/dL, platelets 350 k/mm3, creatinine 0.37 mg/dL.

He had no neurological sequelae related to underlying HUS. He had significant anorexia/nausea throughout the hospitalization and received nasogastric tube feeds until discharge. Stool cultures at the state laboratory were positive for Shiga toxin type 2 (STX2) consistent with likely *E. coli*. An outbreak of infectious diarrhea and HUS was subsequently noted at the patient's daycare, and the local Health Department was closely involved.

**Figure 1:**
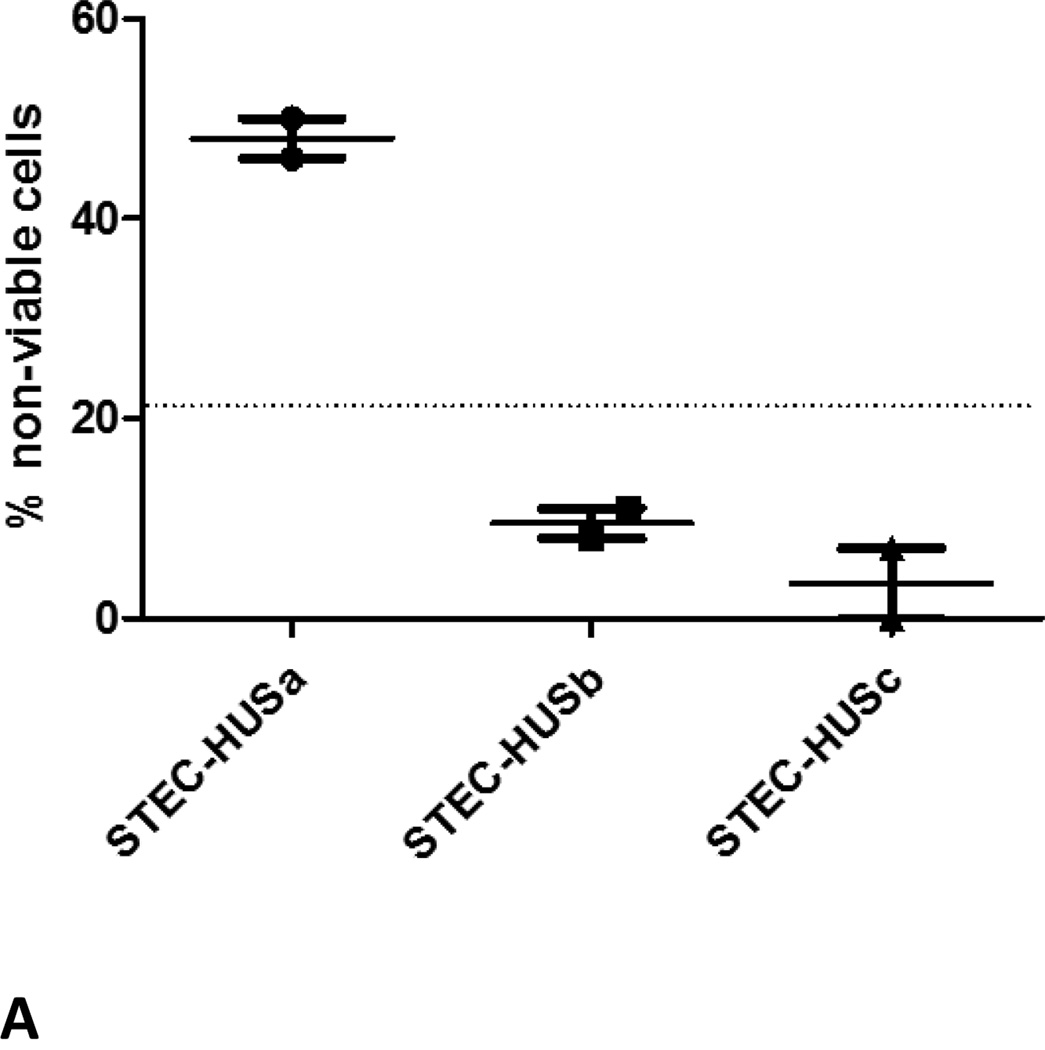

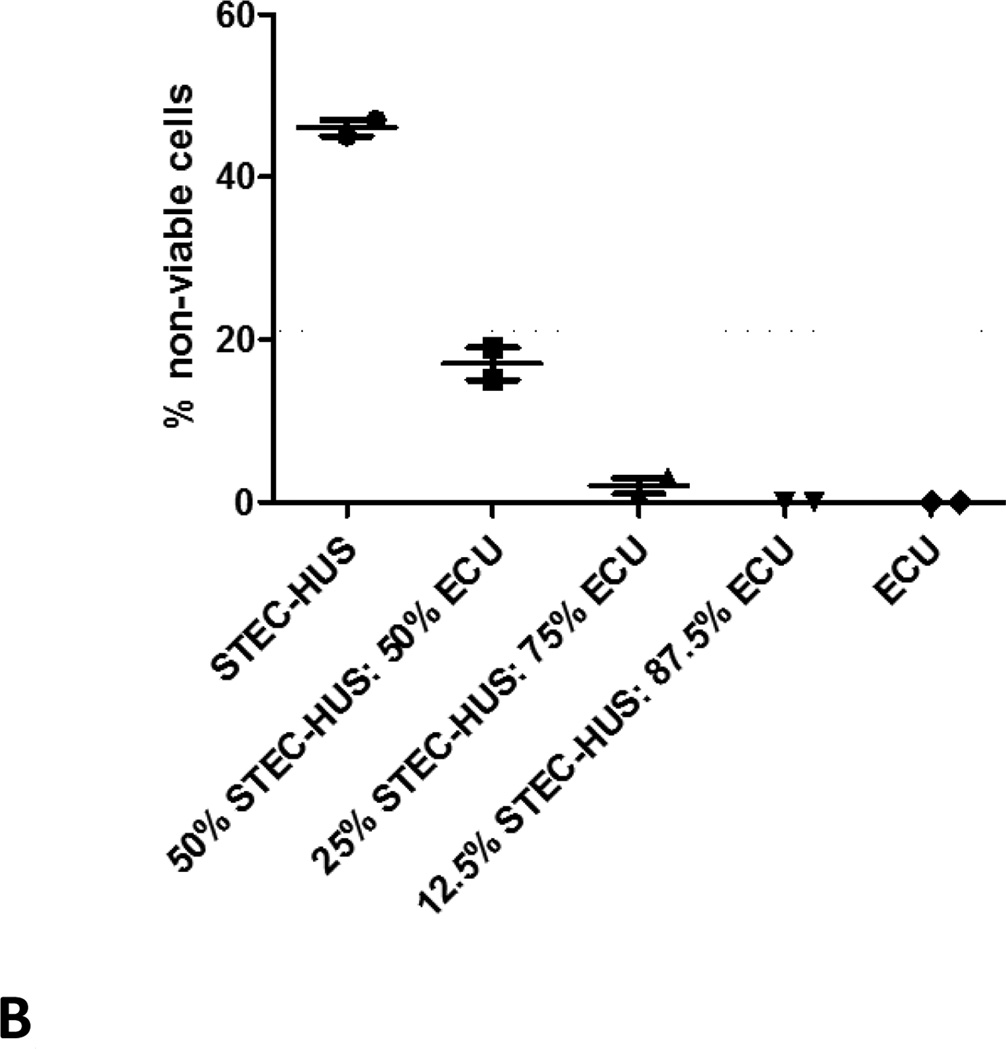

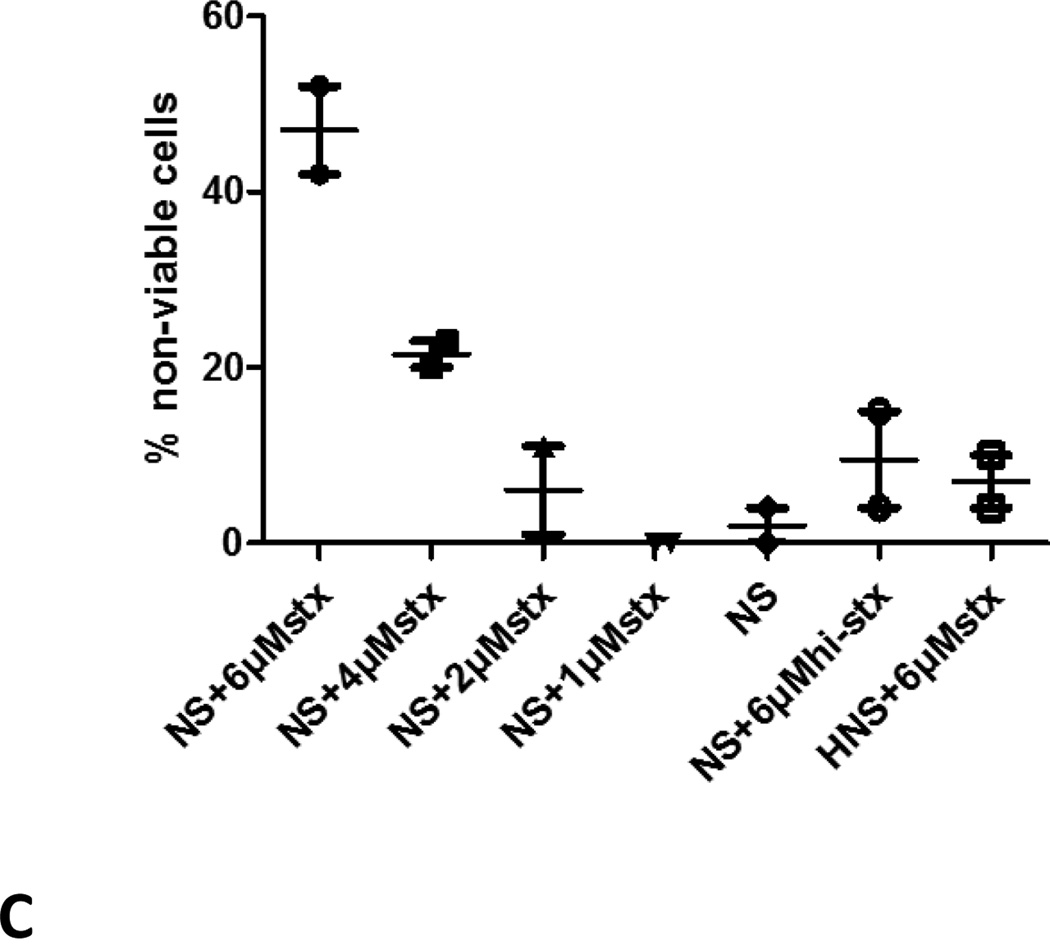
**A):** Complement activation in Shiga-toxin associated HUS- Increased percentage of non-viable cells in the modified Ham test was observed in the acute phase of Shiga-toxin associated HUS (STEC-HUSa) The same patient was tested twice after resolution of the syndrome (4 and 7 days after the first sample, symbolized as STEC-HUSb and c), showing normalization of cell killing. The dotted line symbolizes the cut-off value (21.5% non-viable cells) above which percentage of non-viable cells suggests increased complement activation observed in atypical HUS. Results from two independent experiments are shown. **B):** Complement activation in Shiga-toxin associated HUS- Eculizumab containing serum (ECU) was collected within 60 minutes of eculizumab infusion from a PNH patient. ECU was mixed with serum from the acute STEC-HUS in different percentages (50–50%, 25–75% and 12.5–87.5% of STEC-HUS and ECU sera respectively). Total amount of serum in the assay remained unchanged (20%). Eculizumab containing serum resulted in a normalization of the modified Ham test results in all ratios. Results from two independent experiments are shown. **C):** Complement activation in Shiga-toxin associated HUS- Recombinant shiga-toxin 2 from *E.coli* was added in normal serum (NS) to replicate the effects of STEC-HUS. Addition of shiga-toxin (stx) resulted in increased percentage of non-viable cells compared to normal serum alone and with heat-inactivated shiga-toxin (hi-stx), as well as heat-inactivated normal serum (HNS) with maximum amount of shiga-toxin. Results from two independent experiments are shown.

Further laboratory testing was performed after written informed consent (see Supplementary Methods). Three samples was provided: one in the acute phase of the syndrome and two after the resolution of the syndrome. We utilized the modified Ham test that has been recently described as a functional assay able to detect activation of the alternative pathway of complement observed in complement-mediated hemolytic anemias [10,11]. The test was performed as previously described. Briefly, this rapid and simple cell proliferation assay detects complement-mediated cell killing in the patient serum. As shown in Figure 1A, the acute phase sample caused increased cell killing in the modified Ham test, while the two samples during remission showed normalization of cell killing. These findings suggest that increased complement activation is evident in the acute phase of the disease. To further investigate these findings, we first sought to determine whether complement inhibition with eculizumab nullifies the effects of acute STEC-HUS serum. Therefore, we incubated STEC-HUS serum with serum containing eculizumab at different ratios and tested its effect on the modified Ham test. Indeed, addition of eculizumab containing serum resulted in a normalization of the modified Ham test results in all ratios (Figure 1B).

Then, we tried to mimic the effect caused by STEC-HUS serum utilizing normal serum with Shiga toxin. Recombinant STX2 from *E.coli* was added in normal serum. Addition of Shiga toxin in normal serum resulted in an increase in cell killing (Figure 1C), similar to that caused by STEC-HUS serum. To exclude the possibility of direct Shiga toxin mediated cell lysis, we also tested the effect of Shiga toxin added in complement-inactivated normal serum, which caused no increased in cell killing.

## Discussion

Our report presents the clinical course of a child with STEC-HUS who contracted STEC during a daycare outbreak. He had laboratory findings of increased complement activation in the acute phase of the disease but not after the resolution of the syndrome. In addition, we are able to show that addition of Shiga toxin in normal serum mimics the increased complement activation seen with STEC-HUS serum. More importantly, STECHUS serum is responsive to complement blockade by eculizumab *in vitro*.

Reports on eculizumab treatment in STEC-HUS have been conflicting. In line with our experimental data, initial and later case reports have shown efficacy of early treatment with eculizumab in severe cases of STEC-HUS [6,7]. However, analyses of the German outbreak and registry have not favored the use of eculizumab over other treatment options [8,9]. These analyses are limited by their retrospective nature and lack of randomization that resulted in a comparison between different treatment groups with different disease severities. Our *in vitro* data suggests that activation of the APC plays a role in the end-organ damage of STEC-HUS. It does not prove that eculizumab would alter the natural history of this disease but suggest that if eculizumab therapy is attempted in future clinical trials, it should be administered early in the disease process.

Another interesting observation of these studies was the benefits of antibiotic treatment that resulted in a shorter duration of *E.coli* excretion. Shiga toxin attacks cells expressing globotriosylceramide (Gb3) receptors, such as renal endothelial cells. It has multiple cellular effects that ultimately lead to cellular damage and apoptosis [12]. Several experimental studies have also linked Shiga toxin with complement activation *in vitro*. Indeed, shiga toxin inhibits major complement regulators, complement Factor H (CFH) and CFH-related proteins, mimicking effects caused by the loss-of-function mutations observed in atypical HUS [2,13,14]. Shiga toxin also reduces the expression of other complement regulators, such CD59 and thrombomodulin [15,16]. In addition, shiga toxin seems to directly activate complement component C3 and subsequently, the alternative pathway of complement [17]. Complement activation by shiga toxin has been also suggested by several murine models [3,18,19]. Recently, shiga toxin has also been shown to promote podocyte injury via the alternative complement pathway in an experimental HUS model [20]. These findings correspond to our experimental data showing that the presence of Shiga toxin causes a dose-dependent activation of complement *in vitro*.

In summary, our report suggests that shiga toxin is directly responsible for activating the alternative pathway of complement. Given the epidemic nature of HUS that limits the feasibility of randomized trials, further clinical studies are needed to evaluate early eculizumab treatment in STEC-HUS.

## Supplementary Material

Supplementary methods

## Abbreviations

HD: hospital day
HUS: hemolytic uremic syndrome
PD: peritoneal dialysis
PRBC: packed red blood cell
STEC: Shiga toxin-positive *E. coli*
STX2: Shiga toxin type 2
WBC: white blood cell
