# Supplementary material for: Typical Hus: Evidence of Acute Phase Complement Activation from a Daycare Outbreak
Source: J Clin Exp Nephrol. Author manuscript; Available in PMC 2016 Jul 11. (PMC4940046; doi:10.21767/2472-5056.100011)
Supplement: Supplementary methods [file NIHMS796155-supplement-Supplementary_methods.docx]

**Supplementary Methods**

**Sample collection**

Blood was collected following written informed consent in serum separation tubes and was

centrifuged at 4 oC. Serum was separated and stored at -80 oC. Serum samples were

processed and stored within 4 hours after the blood was drawn to prevent ex vivo

complement activation. The in vitro effects of eculizumab were tested utilizing serum from a

patient treated with eculizumab for paroxysmal nocturnal hemoglobinuria (PNH).

Eculizumab containing serum was drawn following informed consent from a PNH treated

patient within 60 minutes of eculizumab infusion.

**Modified Ham test**

The modified Ham test was performed as previously described (1). Briefly, PNH-like reagent

cells (*PIGA*-null TF-1 cell line) established in our laboratory (2) were plated in U-shaped 96-

well plates at a density of 4,000 cells / well and cultured until confluent. Then, cells were

incubated with 20% of serum in Gelatin Veronal Buffer (GVB, Sigma Aldrich, St Louis, MO) in

triplicates for 30 minutes at 37 oC. Then, the cell proliferation reagent 4-[3-(4-lodophenyl)-2-

(4-nitrophenyl)-2H-5-tetrazolio]-1.3-benzene disulfonate / WST-1 (Roche, Switzerland) was

added for 2 hours at 37 oC. Absorbance was measured in an iMark Microplate Absorbance

Reader (Bio-rad, Hercules, CA) at 490 nm with a reference wavelength at 595 nm. Heatinactivated

serum was used as a negative control. Normal human AB serum (H4522, Sigma-

Aldrich, St Louis, MO) was used as an internal control of the assay. Absorbance values of

each sample were normalized after subtraction of the absorbance value of a blank cell.

Percentage of viable cells was expressed as a ratio of the absorbance of each sample

multiplied by 100, to the absorbance of the same sample’s heat-inactivated control. Thus,

Supplementary files

percentage of non-viable cells (cell killing) was calculated using the following formula: 100 –

(sample’s absorbance * 100 / heat-inactivated sample’s absorbance).

**In vitro effect of eculizumab**

Complement inhibition in vitro was also evaluated using the modified Ham test. Eculizumab

containing serum from a PNH patient was pre-incubated (for 10 minutes at 37 oC) at

different ratios with STEC-HUS serum (50-50%, 25-75% and 12.5-87.5% of STEC-HUS and ECU

serum respectively). Total amount of serum in the assay remained unchanged (20%).

**In vitro effect of shiga-toxin**

Recombinant Shiga toxin 2 from E.coli (List Biological Laboratories, Campbell, CA) at

different concentrations (1,2,4 and 6 μΜ based on previous reports (3)) was pre-incubated

with normal human serum (Sigma-Aldrich, St Louis, MO) at 37 oC for 1 hour. Heat-inactivated

Shiga toxin in normal serum and heat-inactivated normal serum with maximum amounts of

Shiga toxin were used as controls. Serum was then tested in the modified Ham test as

described above.

**References**

1. Gavriilaki E, Yuan X, Ye Z, et al.: Modified Ham test for atypical hemolytic uremic

syndrome. Blood 125: 3637-3646, 2015

2. Savage WJ, Barber JP, Mukhina GL, et al.: Glycosylphosphatidylinositol-anchored

protein deficiency confers resistance to apoptosis in PNH. Experimental hematology 37: 42-

51, 2009

3. Orth D, Khan AB, Naim A, et al.: Shiga toxin activates complement and binds factor

H: evidence for an active role of complement in hemolytic uremic syndrome. J Immunol 182:

6394-6400, 2009
